# Surgical revascularization for severe spasm in the left main coronary artery

**DOI:** 10.1002/ccr3.6815

**Published:** 2023-01-06

**Authors:** Kayo Sugiyama, Masanobu Fujimoto, Hirotaka Watanuki, Katsuhiko Matsuyama

**Affiliations:** ^1^ Department of Cardiac Surgery Aichi Medical University Hospital Nagakute Aichi Japan; ^2^ Department of Cardiology Aichi Medical University Hospital Nagakute Aichi Japan

**Keywords:** carotid artery hypoplasia, coronary artery spasm, endothelial damage

## Abstract

A 46‐year‐old woman who presented with severe stenosis with endothelial damage caused by recurrent spasm in the left main coronary artery received medical therapy. However, she developed severe coronary artery spasm, resulting in circulatory collapse, which was successfully treated with coronary artery bypass grafting.

## Graphical abstract

Surgical revascularization is effective for repeated coronary artery spasms resulting in circulatory collapse. Spasms can sometimes occur in other coronary arteries and may also occur in arteries of other organs.

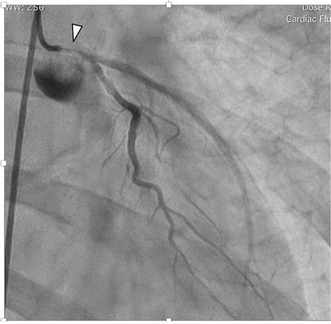

## 1 INTRODUCTION

Variant angina caused by coronary artery spasm can trigger life‐threatening events even with maximally tolerated doses of medication. Coronary artery spasms are associated with adventitial inflammation causing endothelial damage, and once endothelial thickening occurs, it may be refractory to medication.^1^ Both coronary artery bypass grafting (CABG) and percutaneous catheter intervention for variant angina without any atherosclerotic lesions are controversial^2^, ^3^; however, medication cannot be effective in cases with circulatory collapse. Stenting in the coronary artery may provide an effective treatment for persistent severe vasospasm only if other options fail;^3^ however, stenting in the left main trunk is not always safe. Surgical revascularization for variant angina is not a definitive strategy due to problems such as perioperative vasospasm, graft selection, and use of cardiopulmonary bypass. CABG can be a solution in cases of life‐threatening variant angina in the left main coronary artery.^1^, ^3^

## 2 CASE PRESENTATION

The Ethics Committee of Aichi Medical University Hospital approved this case report on Oct 25th, 2021 (Approval Number, 2021–H113). The patient provided written informed consent to publish the details of her case.

**FIGURE 1:**
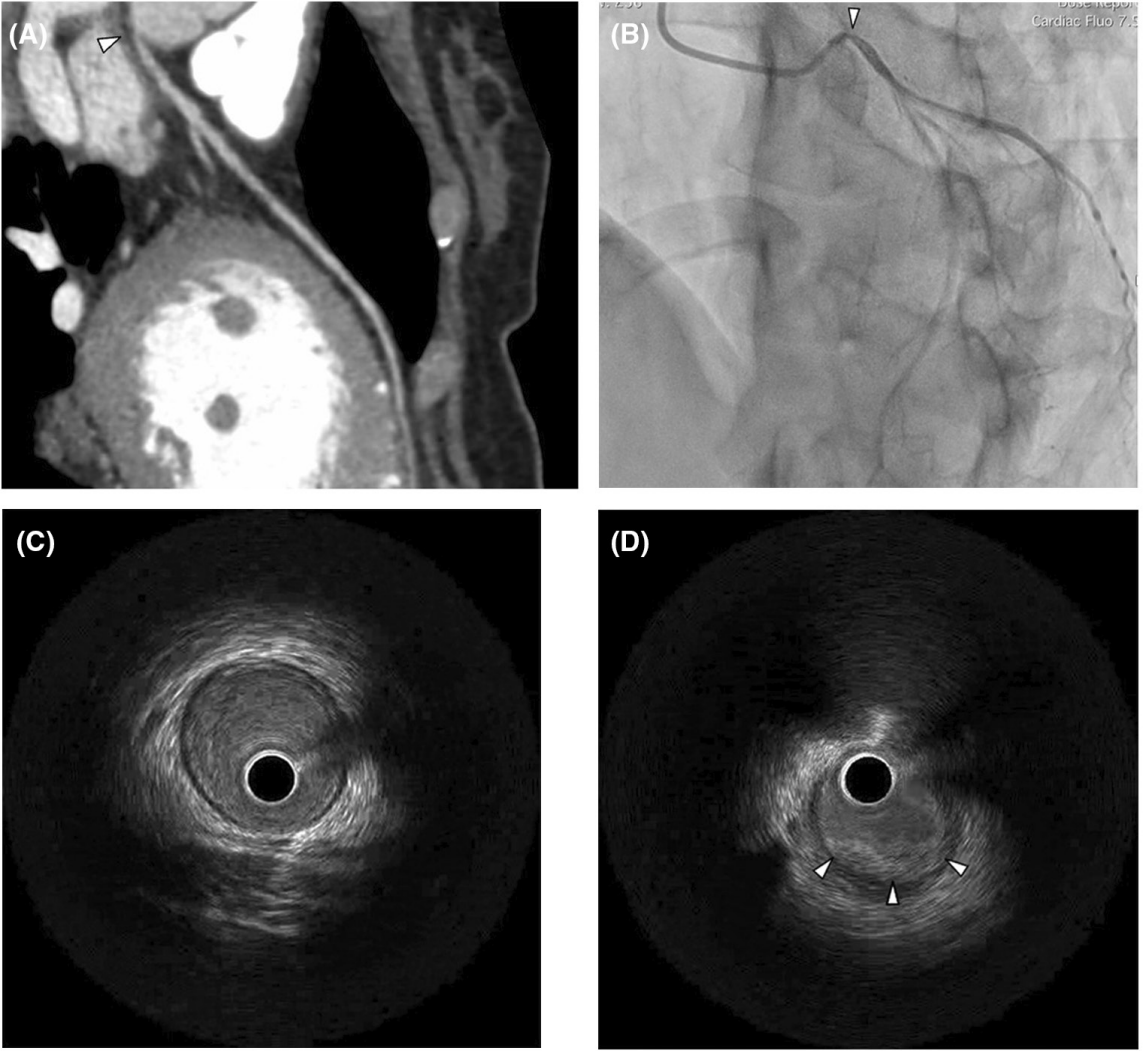
(A) Preoperative multidetector computed tomography showing severe stenosis in the orifice of the left main coronary artery with no evidence of atherosclerotic change (white arrowhead). (B) Preoperative coronary angiography shows severe stenosis of the orifice of the left main coronary artery with no evidence of atherosclerotic change (white arrowhead). (C) Intravascular ultrasound in the non‐stenotic area shows normal intima with adequate diameter. (D) Intravascular ultrasound in the stenotic area shows shrinkage of the wall due to thickened inflamed intima.

A 46‐year‐old woman presented to our hospital with dyspnea. She had previously been treated for hypertension and bronchial asthma at a local hospital. She did no habits related to smoking, alcohol, or drug abuse. Multidetector computed tomography and coronary angiography (Figure 1A,B) revealed severe stenosis of the left main coronary artery orifice with no evidence of atherosclerotic changes. During coronary angiography, intravascular ultrasound showed shrinkage of the wall due to a thickened inflamed intima (Figure 1C,D). She had no obvious history of Kawasaki disease or other inflammatory diseases. Since organic changes in the left main trunk had already developed, as shown by intravascular ultrasound, intracoronary infusion of nitroglycerin was ineffective. As she presented with no remarkable electrocardiographic changes during the exercise stress test, medical therapy with diltiazem hydrochloride (200 mg/day), isosorbide mononitrate (40 mg/day), nicorandil (15 mg/day), nitroglycerin (5 mg/day), aspirin (100 mg/day), fluvastatin sodium (20 mg/day), telmisartan (20 mg/day), and epinastine hydrochloride (20 mg/day) was administered. The patient was asymptomatic until she returned 2 months later.

**FIGURE 2:**
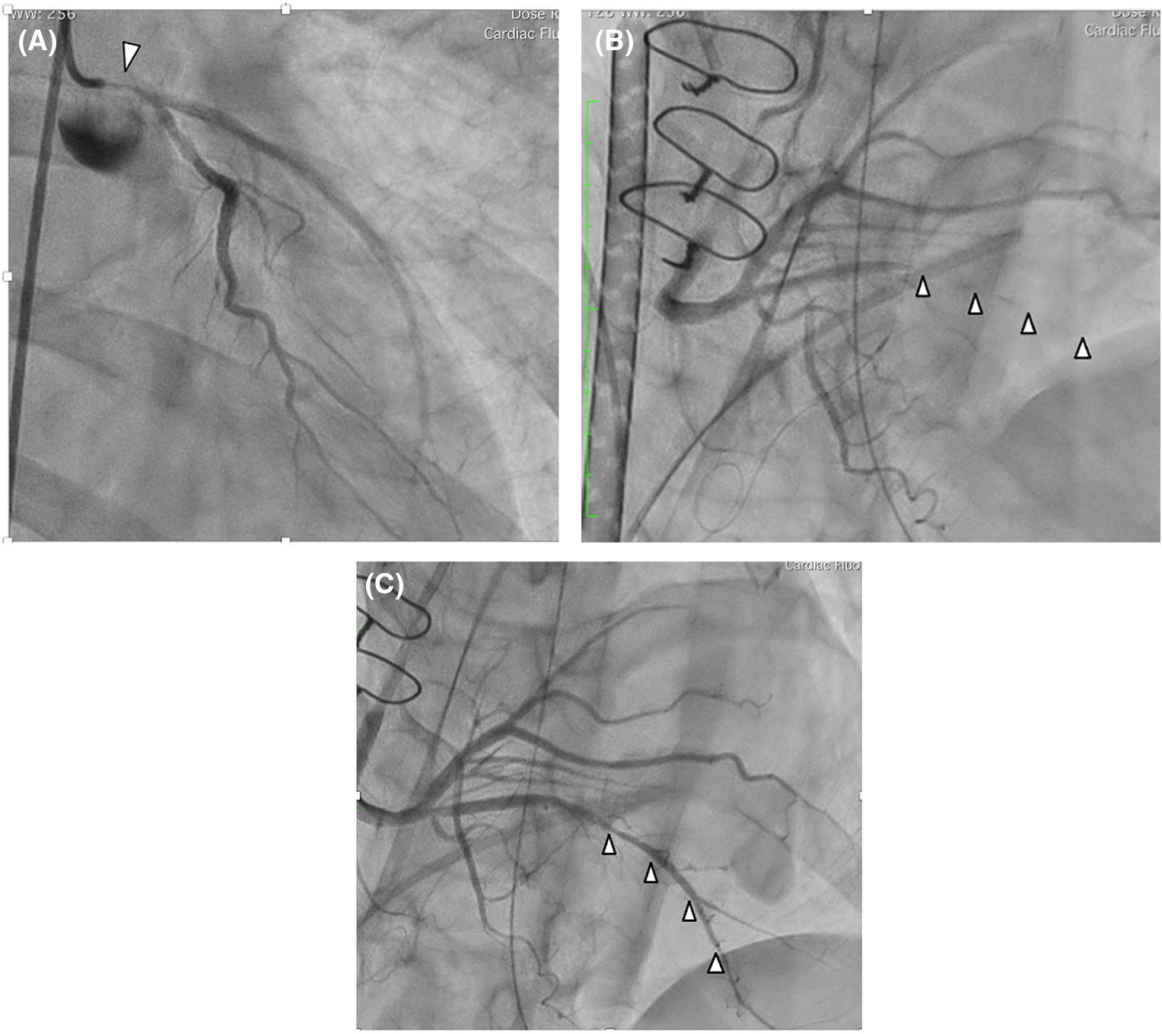
(A) Emergency coronary angiography reveals exacerbation of the severe stenosis in the orifice of the left main coronary artery (white arrowhead). (B) Postoperative urgent coronary angiography demonstrates spastic occlusion of the posterior descending branch (white arrowheads). (C) Postoperative urgent coronary angiography shows released vasospasm after infusion of nitroglycerin and isosorbide dinitrate (white arrowheads).

On the morning of her emergency readmission, she suffered from sudden chest pain resistant to nitroglycerin sublingual tablets and was transferred to our institute. On arrival, she developed ventricular fibrillation that required defibrillation. After establishing extracorporeal membrane oxygenation, emergency coronary angiography revealed worsening of the previous lesion (Figure 2A). Discussion between cardiologists and cardiac surgeons concluded that catheter intervention in the left main trunk seemed unsafe, and CABG seemed to be a solution for this life‐threatening variant angina. Emergency CABG of the left anterior descending artery was performed using the left internal mammary artery, and a saphenous vein graft to the posterolateral branch. Measurements of the intraoperative transit time flow were satisfactory, and no competitive flow was observed in the waveform. During surgery, transesophageal echocardiography revealed a sudden reduction in contraction of the inferior wall, and electrocardiography showed ST depression in lead II. Because hemodynamic instability persisted even after the addition of bypass to the posterolateral branch using a saphenous vein graft, extracorporeal membrane oxygenation was restarted. After surgery, urgent coronary angiography demonstrated patent anastomosis of both bypass grafts but diffuse vasospasm of the posterior descending branch (Figure 2B). Because intravascular ultrasound detected the presence of spasm and infusion of vasodilators via both intravenous and coronary artery orifices was not efficient, a microcatheter with small side holes was passed through the posterior descending branch selectively, resulting in effective resolution of the coronary spasm (Figure 2C). The patient was discharged with medical therapy on postoperative Day 38, following sufficient rehabilitation. The patient remained free from any cardiovascular events for 2 years after surgery.

**FIGURE 3:**
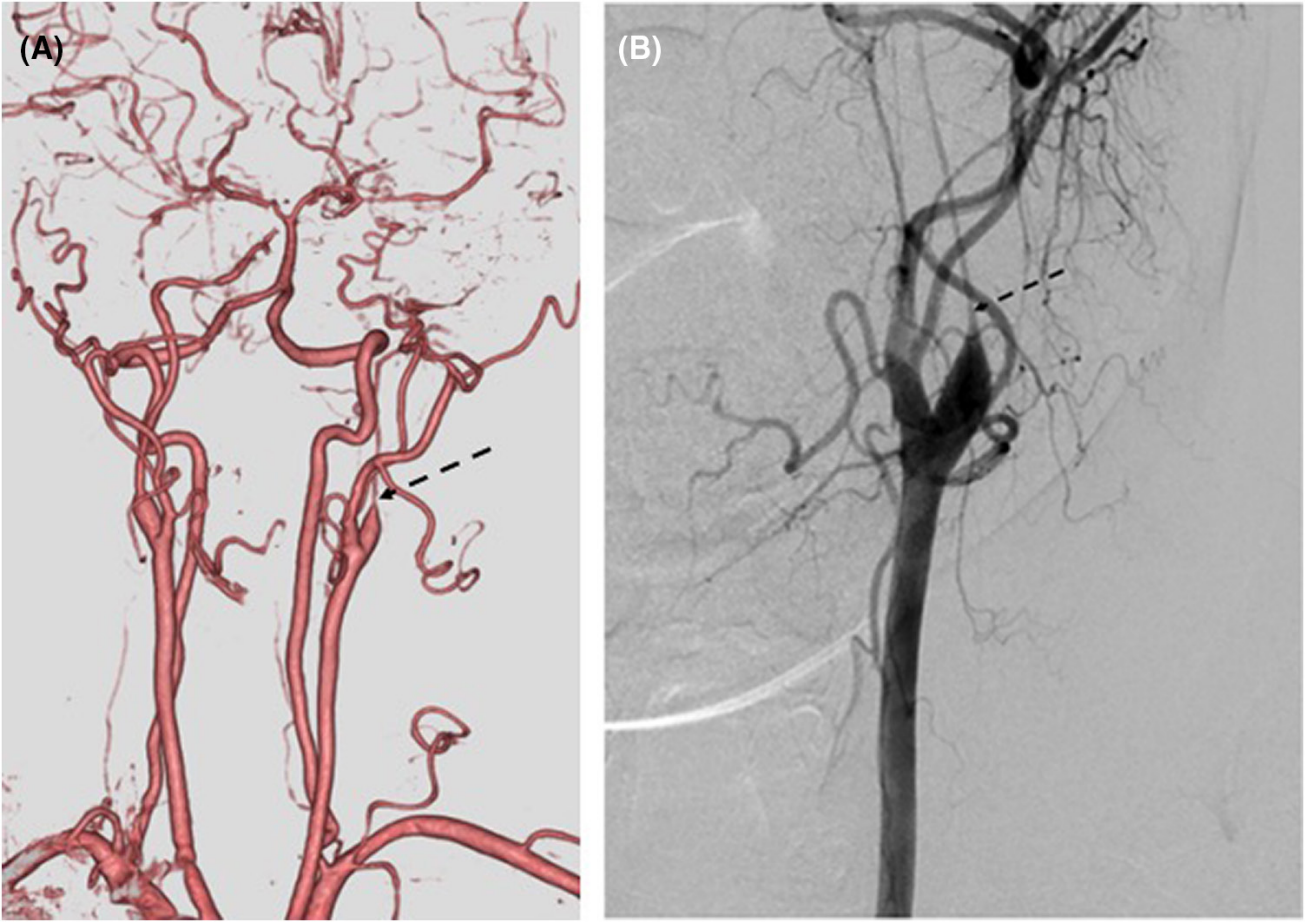
(A) Three‐dimensional head computed tomography indicates severe hypoplasia of the left internal carotid artery (dotted black arrow). (B) Carotid angiography shows a champagne bottle‐neck sign in the proximal portion of the internal carotid artery (dotted black arrow).

During further examination for coronary artery lesions during the previous hospitalization, severe hypoplasia of the left internal carotid artery was detected despite the absence of neurological symptoms (Figure 3A). Carotid angiography revealed a so‐called champagne bottle‐neck sign, which led to speculation that vasospastic occlusion may have caused the hypoplasia (Figure 3B). After discussion with neurosurgeons, revascularization for the lesion was not considered because the hypoplasia had already been completed and there were no symptoms related to cerebrovascular disease due to the intracranial collateral network.

## 3 DISCUSSION

Most patients with coronary artery spasm have a favorable long‐term prognosis and an event‐free clinical course. However, a few patients sustain life‐threatening events, even with maximally tolerated doses of medication.^1^ Coronary artery spasms are associated with adventitial inflammation. Shimokawa et al. suggested that under long‐term stimulation of the adventitia with some inflammatory cytokines, smooth muscle phenotypes are altered, resulting in a coronary vasospastic response and slight neointimal formation.^2^ Irreversible changes caused by endothelial damage can be resistant to optimal medical treatment. Although the patient in the present case was treated with optimal medication, including coronary vasodilators, calcium antagonists, antiplatelets, and statins, she developed circulatory collapse due to spasm in the left main coronary artery. Racial differences between Caucasians and Japanese individuals with regard to coronary vasomotor reactivity are controversial. Some reports suggest that coronary artery spasm is more frequently recognized in Japanese people than in Caucasians,^4^, ^5^ while others have found no significant differences between races.^6^

Ono et al. stated that the surgical method is indicated for patients with medically intractable, life‐threatening variant angina as a last resort.^1^ Saxena et al. reported that coronary artery and graft stenting might provide an effective treatment when other options fail.^3^ Spasm occurred at the edge of the implanted stent in 28% of patients treated with intracoronary stenting.^7^ Conversely, the experience of Pasternak et al. suggests that an explanation for the success of CABG is directly related to the severity of the fixed stenosis present.^8^ Sussman et al. reported successful CABG in two patients who had focal coronary spasm with fixed obstructions of less than 20%.^9^ Surgical methods are indicated for patients with medically intractable, life‐threatening variant angina as a last resort.^1^ Because the present case showed fixed stenosis due to repeated inflammatory changes in the left main coronary artery, circulatory collapse refractory to full medication, and catheter intervention seemed not to be suitable, surgical revascularization was chosen.

Ono et al. proposed that the internal mammary artery would be a more suitable graft for patients with variant angina.^1^ In the revascularization reported by Sussman et al., a saphenous vein bypass graft was placed distal to the area of focal spasm and the native coronary artery was ligated proximally.^9^ In the present case, the internal mammary artery and saphenous vein graft were chosen because they are less likely to cause spasm compared to the radial artery or gastroepiploic artery.^10^ Several factors thought to provoke spasm may interact in the postoperative period, including high endogenous catecholamine levels, physical manipulation of a coronary artery during dissection for placement of a bypass graft, and various mediators released during inflammatory response.^11^ In the present case, intravenous infusion of vasodilator was not effective, and direct intracoronary infusion of a combination of nitroglycerin and isosorbide dinitrate was effective for diffuse vasospasm of the posterior descending branch. Ono et al. were also concerned that coronary spasm during the anastomosis procedure would be devastating; therefore, they performed the surgery with the use of cardiopulmonary bypass.^1^ We adopted CABG with cardiopulmonary bypass for the same reason.

It is unclear how a spasm in the coronary artery is associated with spasm in other non‐coronary arteries.^12^ There have been some reports that describe idiopathic carotid artery and coronary artery vasospasm;^13^ however, this phenomenon is limited to case reports, and the cause is still unknown. In the present case, it was speculated that severe hypoplasia of the carotid artery was caused by vasospastic occlusion since carotid angiography presented a champagne bottle‐neck sign representing vasospastic occlusion. Further studies on this relationship are required. If spasm occurs in the carotid artery, the subclavian and internal mammary arteries could also develop spasms. A close follow‐up with meticulous medical therapy is necessary.

## 4 CONCLUSION

Surgical revascularization is effective for endothelial damage due to repeated coronary artery spasms, resulting in circulatory collapse. The occurrence of vasospasm in the uninvolved coronary artery should be considered during revascularization, and regular follow‐up with optimal medication is essential. Further studies on the relationship between spasms in the coronary arteries and other arteries are warranted.

## AUTHOR CONTRIBUTIONS

KS involved in analysis, data interpretation, and drafting and revising manuscript. MF involved in analysis and data interpretation. KM involved in analysis, drafting and revising manuscript. All authors involved in final approval of the paper, agreement to be accountable for the integrity of the case reports.

## CONFLICT OF INTEREST

None declared.

## ETHICAL APPROVAL

The Ethics Committee of Aichi Medical University Hospital approved this case report on October 25, 2021 (Approval Number, 2021–H113).

## CONSENT

Written informed consent was obtained from the patient to publish this report in accordance with the journal's patient consent policy.

## Data Availability

Data openly available in a public repository that issues datasets with https://doi.org/10.1002/ccr3.6815.
